# Supplementary material for: Consistent Effects of Whey Protein Fortification on Consumer Perception and Liking of Solid Food Matrices (Cakes and Biscuits) Regardless of Age and Saliva Flow
Source: Foods. 2020 Sep 21;9(9):1328. doi: 10.3390/foods9091328 (PMC7555850; doi:10.3390/foods9091328)
Supplement: Supplementary file 1 [file foods-09-01328-s001.pdf]

Supplementary Materials: The following are available online at www.mdpi.com/xxx/s1, Figure S1. Additional factors influencing older adults salivary flow rates (mL/min) in both studies. Figure S2. Histogram of older adults unstimulated saliva flow data in both studies. Figure S3: Volunteers attribute perception mean ratings of cakes and biscuit by saliva flow (SF) (pilot study). Figure S4: Volunteers attribute perception mean ratings of cupcakes by saliva flow (SF) (main study). Table S1: Cake and biscuit (g per 100 g) ingredients formations in both studies. Table S2: Additional factors influencing volunteers liking, easiness to eat and swallow, attribute perception, appropriateness of attribute level (Just-About-Right) and appetite of product in both studies. Table S3: Volunteer counts of cake and biscuit preference in the pilot study. Table S4: Summary of volunteers comments in both studies

Age was significantly associated ($p < 0.0001$) with medication in both studies, where only older adults reported regular medication use (Table 5 in the results section). However, the influence of medication on saliva flow in older adults varied between the studies as outlined in Figure S1. Accordingly, histogram analysis was carried out to understand better the distribution of the data and this revealed data in the pilot study was centred more towards the left-hand side and more spread over a greater range, whilst in the main study the data was more compact within a similar range, as demonstrated in Figure S2. Therefore, it could be suggested that volunteers lacked experience in saliva collection hence the lower saliva flow rates whereas in the main study volunteers were more familiar with saliva collection leading to higher salivary flow rates and a proposed rationale for a significant effect of medication on unstimulated saliva flow in the main study. As highlighted in our previous work [17] if volunteers are unfamiliar with saliva collection, familiarisation session could be beneficial. It should be noted that each volunteer's medication was screened for potential side effects likely to influence saliva flow, therefore it was considered medication was unlikely to have caused an increase in saliva flow in this case. In addition there was an imbalance of numbers of volunteers taking medication in the main study compared with the pilot study. During the pilot study less than half (19 out of 42 older adults) of the volunteers were taking medication, whereas in the main study it was more than half (19 out of 32 older adults).

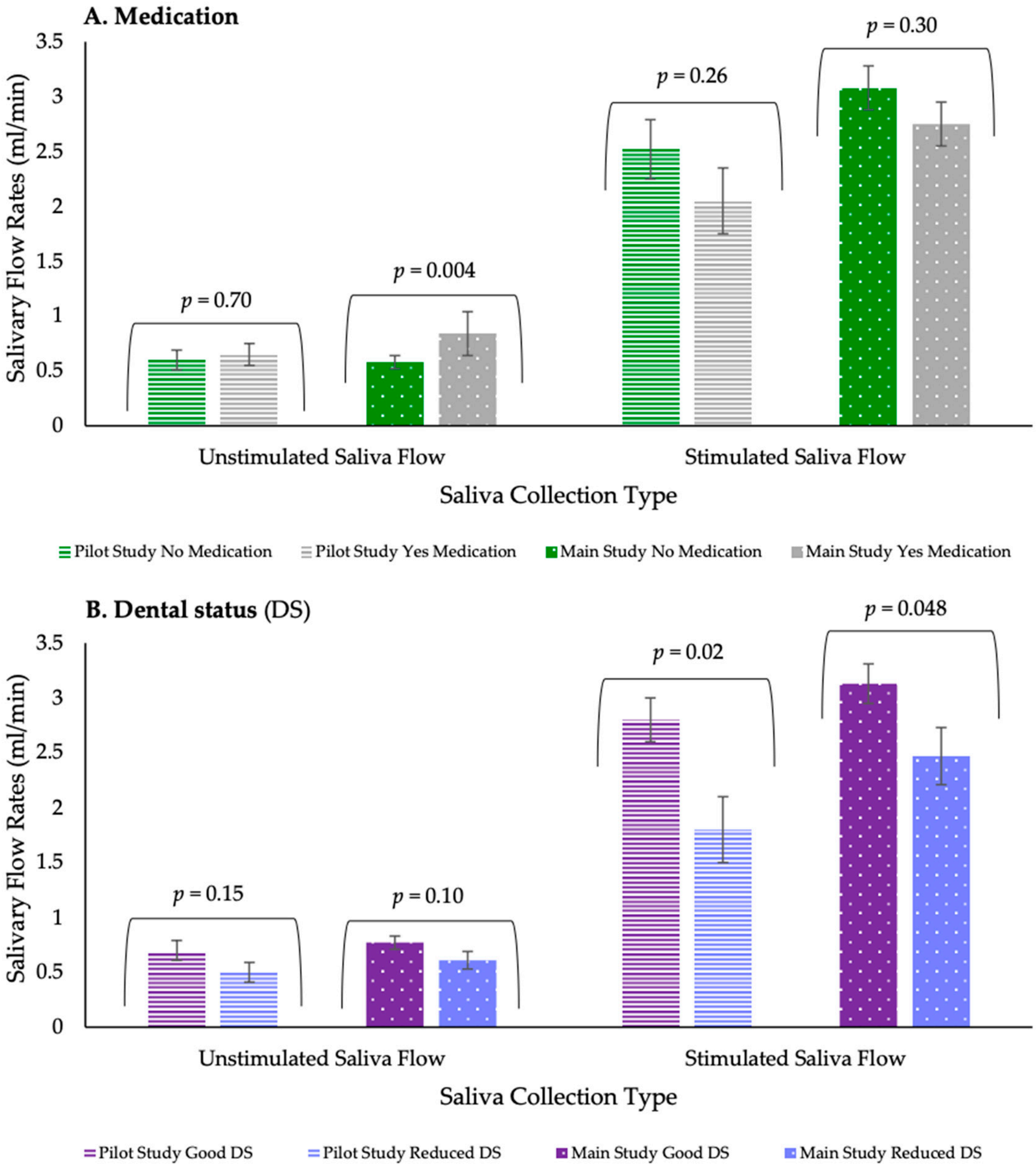

Figure S1. Additional factors influencing older adults salivary flow rates (mL/min) in both studies. Values are expressed as LSM estimates ± standard error from SAS output. Significant differences ($p < 0.05$) were reported between groups with relevant p value above each group.

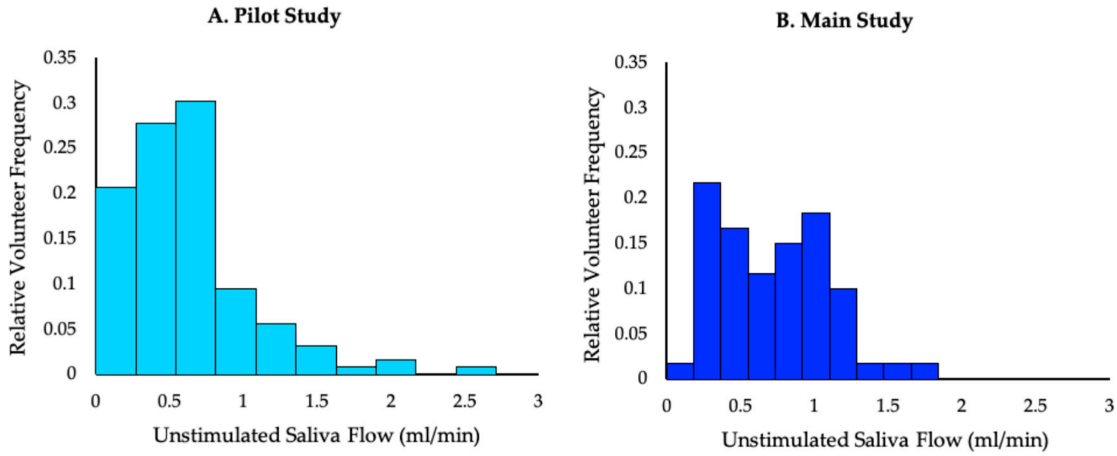

Figure S2. Histograms of older adults unstimulated saliva flow data in both studies.

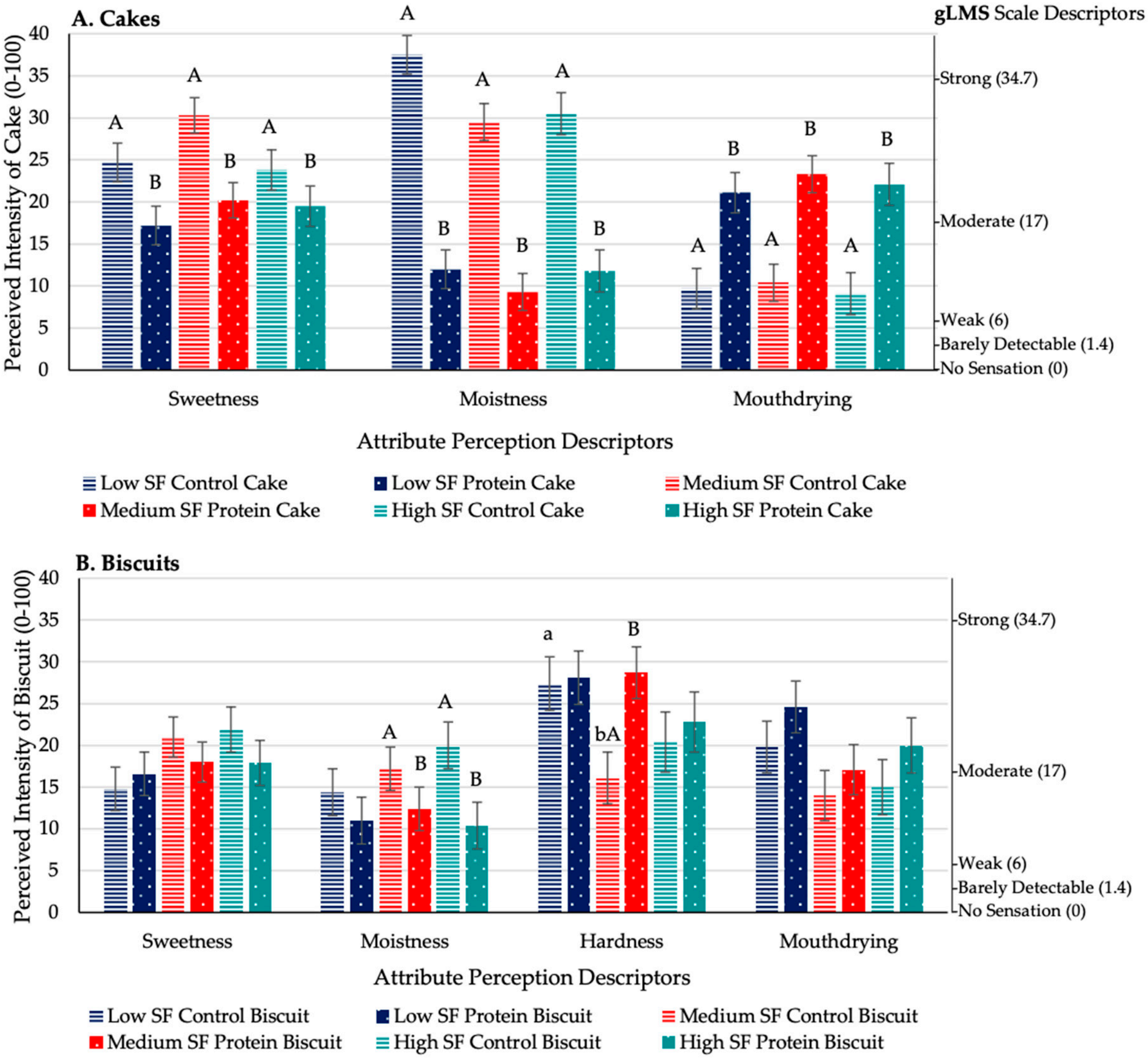

Figure S3. Volunteers attribute perception mean ratings of cakes and biscuits by saliva flow (SF) (pilot study: n = 84; gLMS antilogged data, scale 0–100 summarised on the right of the figure). Values are expressed as LSM estimates ± standard error from SAS output. Significant differences ($p < 0.05$) between saliva flow groups within sample type are denoted by differing small letters and significant differences between samples within saliva flow groupings are denoted by differing capital letters; no letter reflects no significance difference. Individual saliva flow groupings are derived from unstimulated saliva flow only, through tertiary analysis.

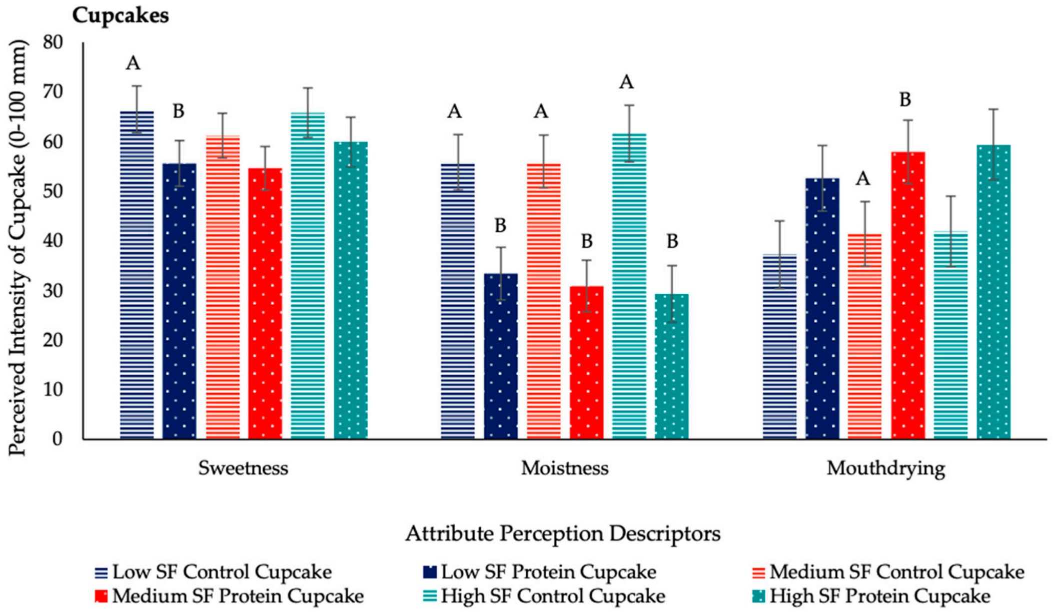

Figure S4. Volunteers attribute perception mean ratings of cupcakes by saliva flow (SF) (main study: *n* = 70; VAS, 0–100 mm). Values are expressed as LSM estimates ± standard error from SAS output. Significant differences ($p < 0.05$) between saliva flow groups within sample type are denoted by differing small letters and significant differences between samples within saliva flow groupings are denoted by differing capital letters; no letter no significance difference. Individual saliva flow groupings are derived from unstimulated saliva flow only, through tertiary analysis.

Table S1. Cake and biscuit (g per 100 g) ingredients formations in both studies.

Ingredients	Control Cake	Protein Cake	Control Biscuit	Protein Biscuit	Control Cupcake	Protein Cupcake
Sainsburys Self Raising Flour	21.9	20.6	-	-	23.0	23.0
Dr Oetker Baking Powder	1.1	1.0	0.8	0.8	-	-
Sainsburys Woodland Free-Range Eggs	21.9	20.6	-	-	18.6	18.6
Sainsburys British Whole Milk	9.8	9.2	-	-	5.0	5.0
Sainsburys English Unsalted Soft Butter	21.9	20.6	-	-	23.0	23.0
Sainsburys White Caster Sugar	21.9	20.6	-	-	23.0	23.0
Dr Oetker Madagascan Vanilla Extract	1.1	1.0	-	-	-	-
Volac Whey Protein Isolate	-	6.1	-	7.5	-	-
Volac Whey Permeate	-	-	-	-	6.6	-
Volac Whey Protein Concentrate	-	-	-	-	-	6.6
Lemon Zest (Sainsburys unwaxed lemons)	-	-	2.1	1.9	0.8	0.8
Silbury Cream 64	-	-	26.9	24.9	-	-
Sainsburys Light Soft Brown Sugar	-	-	21.3	19.6	-	-
Sainsburys British Plain Flour	-	-	19.9	18.4	-	-
Sainsburys Scottish Porridge Oats	-	-	19.9	18.4	-	-
Water	-	-	8.5	7.9	-	-
Dr Oetker Glycerine	-	-	0.3	0.3	-	-

Table S2. Additional factors influencing volunteers liking, easiness to eat and swallow, attribute perception, appropriateness of attribute level (Just-About-Right) and appetite of products in both studies (PS[1]: pilot study; MS[2]: main study).

	Medication		Dental Status		Mouth Behaviour			Sex		Visit	
	No	Yes	Good	Reduced	Chewer	Cruncher	Other	Male	Female	One	Two
	PS (*n* = 65)	PS (*n* = 19)	PS (*n* = 64)	PS (*n* = 20)	PS (*n* = 42)	PS (*n* = 33)	PS (*n* = 9)	PS (*n* = 31)	PS (*n* = 53)	PS (*n* = 84)	PS (*n* = 82)
	MS (*n* = 52)	MS (*n* = 18)	MS (*n* = 60)	MS (*n* = 10)	MS (*n* = 29)	MS (*n* = 25)	MS (*n* = 13)	MS (*n* = 27)	MS (*n* = 43)	MS (n/a)	MS (n/a)
**Appearance Liking**											
Cake[1]	6.7 ± 0.1	6.8 ± 0.2	6.8 ± 0.1	6.8 ± 0.2	6.9 ± 0.1	6.3 ± 0.2*	7.0 ± 0.3	6.9 ± 0.2	6.8 ± 0.2	6.8 ± 0.1	6.8 ± 0.1
Biscuit[1]	5.8 ± 0.2	5.7 ± 0.4	5.9 ± 0.2	5.6 ± 0.3	6.0 ± 0.2	5.3 ± 0.2*	5.9 ± 0.4	5.3 ± 0.2	6.1 ± 0.2	-	-
Cupcake[2]	63.1 ± 5.2	54.9 ± 5.4	60.1 ± 3.5	57.9 ± 7.1	54.1 ± 4.1	62.0 ± 4.6	60.9 ± 5.5	61.3 ± 4.4	56.8 ± 3.9	-	-
**Overall Liking**											
Cake[1]	6.2 ± 0.2	5.4 ± 0.3*	5.7 ± 0.2	5.9 ± 0.3	6.0 ± 0.2	5.7 ± 0.2	5.7 ± 0.4	5.9 ± 0.2	5.8 ± 0.2	5.8 ± 0.2	5.9 ± 0.2
Biscuit[1]	5.9 ± 0.2	5.7 ± 0.2	5.6 ± 0.2	5.9 ± 0.3	5.7 ± 0.2	5.6 ± 0.3	6.1 ± 0.5	5.7 ± 0.2	5.8 ± 0.3	-	-
Cupcake[2]	59.9 ± 5.7	56.7 ± 5.8	65.5 ± 3.8	51.1 ± 7.8*	50.4 ± 4.5	61.7 ± 5.0	62.8 ± 6.0	60.2 ± 4.9	56.3 ± 4.2	-	-
**Easiness to Eat**											
Cake[1]	3.8 ± 0.1	3.7 ± 0.1	3.7 ± 0.1	3.8 ± 0.1	3.7 ± 0.1	3.7 ± 0.1	3.8 ± 0.2	3.7 ± 0.1	3.8 ± 0.1	3.8 ± 0.1[a]	3.6 ± 0.1[b]
Biscuit[1]	3.6 ± 0.1	3.2 ± 0.2	3.5 ± 0.1	3.3 ± 0.2	3.4 ± 0.1	3.3 ± 0.1	3.5 ± 0.2	3.3 ± 0.1	3.4 ± 0.1	-	-
Cupcake[2]	56.5 ± 5.3	60.1 ± 5.4	73.0 ± 3.5	43.6 ± 7.1*	52.7 ± 4.1	60.4 ± 4.6	61.8 ± 5.5	55.6 ± 4.5	61.0 ± 3.9	-	-
**Easiness to Swallow**											
Cake[1]	3.6 ± 0.1	3.4 ± 0.2	3.4 ± 0.1	3.5 ± 0.2	3.6 ± 0.1	3.4 ± 0.1	3.4 ± 0.2	3.4 ± 0.1	3.5 ± 0.1	3.6 ± 0.1[a]	3.3 ± 0.1[b]
Biscuit[1]	3.6 ± 0.1	3.2 ± 0.2	3.4 ± 0.1	3.4 ± 0.2	3.4 ± 0.1	3.3 ± 0.1	3.5 ± 0.2	3.2 ± 0.1	3.6 ± 0.1	-	-
Cupcake[2]	54.2 ± 4.7	57.9 ± 4.9	68.6 ± 3.1	43.6 ± 6.4*	50.5 ± 3.7	57.5 ± 4.1	60.1 ± 4.9	52.3 ± 4.0	59.8 ± 3.4	-	-
**Sweetness**											
Cake[1]	21.0 ± 1.4	24.3 ± 2.5	21.7 ± 1.6	23.5 ± 2.3	21.1 ± 1.7	22.3 ± 1.8	24.4 ± 3.1	22.0 ± 2.0	23.2 ± 1.8	22.6 ± 1.7	22.7 ± 1.7
Biscuit[1]	18.3 ± 1.5	18.4 ± 2.7	19.9 ± 1.7	16.9 ± 2.5	16.6 ± 1.8	19.9 ± 2.0	18.7 ± 3.3	18.2 ± 2.1	18.5 ± 1.9	-	-
Cupcake[2]	65.0 ± 4.4	56.2 ± 4.6	61.6 ± 3.0	59.7 ± 6.0	56.2 ± 3.5	62.0 ± 3.9	63.6 ± 4.7	62.0 ± 3.8	59.1 ± 3.3	-	-
**Moistness**											
Cake[1]	22.4 ± 1.4	21.5 ± 2.5	22.8 ± 1.6	20.8 ± 2.3	23.3 ± 1.7	21.0 ± 1.8	20.9 ± 2.9	22.1 ± 2.0	21.4 ± 1.7	21.9 ± 1.7	21.7 ± 1.7
Biscuit[1]	14.6 ± 1.7	14.0 ± 2.9	14.9 ± 1.8	13.7 ± 2.7	11.6 ± 2.0	14.2 ± 2.1	17.0 ± 3.7	14.4 ± 2.2	14.0 ± 2.0	-	-
Cupcake[2]	49.6 ± 4.7	39.3 ± 4.9	43.3 ± 3.1	45.7 ± 6.4	44.4 ± 3.7	43.6 ± 4.1	45.6 ± 4.9	44.7 ± 4.0	44.3 ± 3.4	-	-
**Mouthdrying**											
Cake[1]	13.2 ± 1.4	18.8 ± 2.5*	18.1 ± 1.6	13.9 ± 2.3	15.7 ± 1.7	15.7 ± 1.8	16.7 ± 3.1	17.3 ± 2.0	14.6 ± 1.8	15.7 ± 1.7	16.3 ± 1.7
Biscuit[1]	20.5 ± 2.0	16.4 ± 3.4	20.5 ± 2.1	16.8 ± 3.2	18.4 ± 2.3	17.5 ± 2.5	19.4 ± 4.2	18.7 ± 2.7	18.2 ± 2.4	-	-

Cupcake[2]	43.3 ± 6.0	53.4 ± 6.1	44.8 ± 4.0	52.0 ± 8.1	48.4 ± 4.7	46.8 ± 5.2	50.0 ± 6.2	54.4 ± 5.1	42.3 ± 4.4		
**Hardness**											
Biscuit[1]	22.8 ± 1.9	25.1 ± 3.1	25.6 ± 2.0	22.2 ± 3.0	25.3 ± 2.2	22.7 ± 2.3	23.8 ± 4.3	24.0 ± 2.6	23.8 ± 2.4	-	-
**JAR Flavour**											
Cake[1]	2.7 ± 0.07	2.6 ± 0.1	2.6 ± 0.08	2.7 ± 0.1	2.7 ± 0.08	2.8 ± 0.08	2.7 ± 0.1	2.7 ± 0.09	2.7 ± 0.08	2.7 ± 0.08	2.7 ± 0.08
Biscuit[1]	2.6 ± 0.09	2.6 ± 0.1	2.7 ± 0.1	2.5 ± 0.1	2.4 ± 0.1	2.7 ± 0.1	2.8 ± 0.1	2.6 ± 0.1	2.7 ± 0.1	-	-
**JAR Colour**											
Cake[1]	2.9 ± 0.03	2.9 ± 0.06	2.9 ± 0.04	3.0 ± 0.06	3.0 ± 0.04	2.9 ± 0.04	2.8 ± 0.08	2.9 ± 0.04	2.9 ± 0.04	2.9 ± 0.04	2.9 ± 0.04
Biscuit[1]	2.9 ± 0.08	2.8 ± 0.1	2.9 ± 0.09	2.8 ± 0.1	2.8 ± 0.1	3.0 ± 0.1	2.9 ± 0.1	2.9 ± 0.1	2.9 ± 0.1	-	-
**Appetite** (cupcakes only)											
Hungry[2]	−13.8 ± 4.7	−10.1 ± 5.0	-14.7 ± 3.1	−9.2 ± 6.4	−10.3 ± 3.7	−14.0 ± 4.2	−11.5 ± 4.9	−12.6 ± 4.1	−11.3 ± 3.4	-	-
Thirsty[2]	12.0 ± 5.2	13.0 ± 5.5	6.5 ± 3.4	18.4 ± 7.2	11.3 ± 4.1	11.0 ± 4.8	14.9 ± 5.4	13.0 ± 4.6	11.9 ± 3.9	-	-
Desire[2]	−12.8 ± 4.7	−21.1 ± 5.0	−22.1 ± 3.1	−11.9 ± 6.6*	−17.5 ± 3.7	−12.7 ± 4.2	−20.7 ± 4.9*	−13.1 ± 4.1	−20.8 ± 3.5*	-	-
Satiety[2]	8.6 ± 5.3	5.6 ± 5.6	13.4 ± 3.5	0.82 ± 7.3	6.8 ± 4.2	6.0 ± 4.8	8.6 ± 5.6	1.3 ± 4.7	12.9 ± 4.0	-	-
Fullness[2]	10.8 ± 4.9	7.0 ± 5.1	14.0 ± 3.2	3.9 ± 6.8	9.7 ± 3.9	9.8 ± 4.4	7.1 ± 5.1	5.5 ± 4.3	12.2 ± 3.6	-	-
Prospective Consumption[2]	−5.5 ± 4.1	−4.5 ± 4.3	−11.4 ± 2.8	−1.4 ± 5.7*	−4.6 ± 3.2	−3.6 ± 3.8	−6.8 ± 4.3	−0.29 ± 3.7	−10.2 ± 3.0	-	-

Values are expressed as LSM estimates ± standard error from SAS output. Significant differences ($p < 0.05$) between categories are denoted by differing letters. Pilot study[1] (PS) $n = 84$; cake and biscuit liking, easiness to eat and swallow and JAR measured on a 9-point and 5-point scale and attribute perception was measured on a gLMS logarithmic scale (antilogged values 0–100 scale presented) and main study[2] (MS) $n = 70$; cupcakes were measured on a VAS 0–100 mm and appetite ratings reflect a change from baseline (positive/negative values relate to the specific appetite rating being measured, for example, a negative hunger rating represents a decline in hunger). Visit only applied to the pilot study cakes and appetite only applied to cupcakes during the main study only. Mouth behaviour 'other' reflects smooshers/sucker in the pilot study and smooshers in the main study.

Table S3. Volunteer counts of cake and biscuit preference in the pilot study.

	**Cake**		Significance of sample (*p* value)	**Biscuit**		Significance of sample (*p* value)
	Control	Protein		Control	Protein	
Total (n = 84)	144	22	**< 0.0001**	52	32	**0.02**
Younger adults (n = 42)	66	16	**< 0.0001**	22	20	0.44
Older adults (n = 42)	78	6	**< 0.0001**	30	12	**0.004**

Cakes were measured in duplicate at visit one and two (n = 166; YA: n = 82; OA: n = 84 (where 2 YA dropped out after visit one)).

Table S4. Summary of volunteers comments in both studies.

	**Flavour Related Comments**									**Texture Related Comments**								
	Positive			Negative			No Comments Provided			Positive			Negative			No Comments Provided		
	Total	YA	OA	Total	YA	OA	Total	YA	OA	Total	YA	OA	Total	YA	OA	Total	YA	OA
Control Cake[1]	85	33	52	26	11	15	55	38	17	101	38	63	17	10	7	48	34	14
Protein Cake[1]	29	17	12	81	26	55	57	39	18	32	7	25	94	52	42	40	24	19
Control Biscuit[1]	34	10	24	22	14	8	28	10	18	38	14	24	14	7	7	32	21	11
Protein Biscuit[1]	9	2	7	46	22	23	29	12	17	18	9	9	33	14	19	33	19	14
Control Cupcake[2]	59	33	26	4	1	3	3	2	1	34	19	15	28	14	14	4	3	1
Protein Cupcake[2]	40	26	14	23	7	16	4	4	0	20	13	7	42	19	23	5	5	0

Positive refers to good, JAR, nice, pleasant, tasty, fresh, soft, vanilla or lemony flavour and negative refers to weak flavour, dry, disliked, hard, rough, coarse, dense and sticky. Pilot study[1] (cakes and biscuits) n = 84; younger adults (YA): n = 42; older adults (OA): n = 42 and main study[2] (cupcakes) n = 70; YA: n = 38; OA: n = 32. During the pilot study[1]; cakes were measured in duplicate at visit one and two (n = 166; OA: n = 84; YA: n = 82 (where 2 YA dropped out after visit one)).
